# Supplementary material for: Glycolysis downregulation is a hallmark of HIV‐1 latency and sensitizes infected cells to oxidative stress
Source: EMBO Mol Med. 2021 Jul 20;13(8):e13901. doi: 10.15252/emmm.202013901 (PMC8350904; doi:10.15252/emmm.202013901)
Supplement: Supplementary file 1 — Expanded View Figures PDF [file EMMM-13-e13901-s001.pdf]

Expanded View Figures

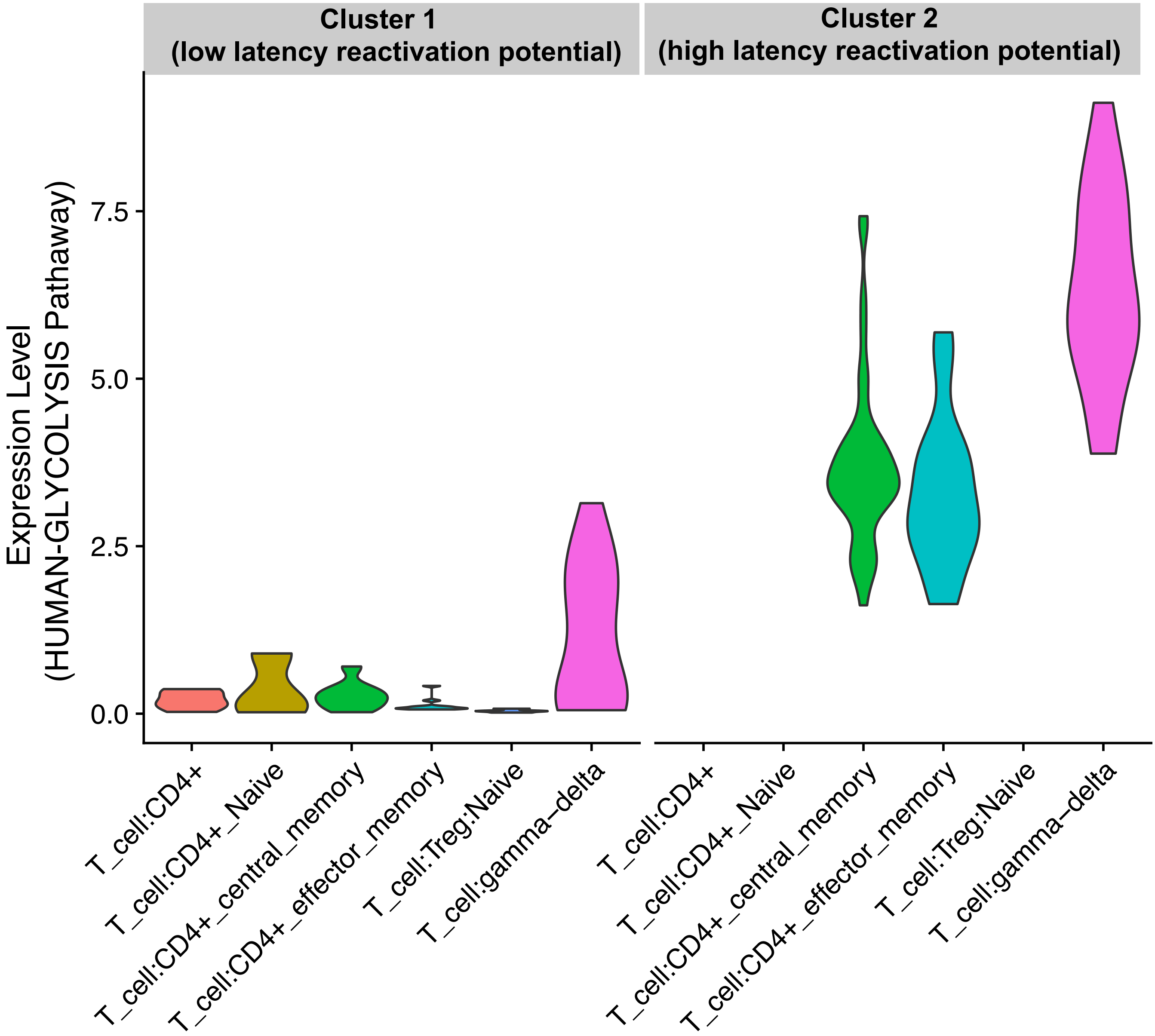

Figure EV1. Single-cell transcriptional modulation of glycolytic enzymes in CD4+ T-cell subsets.

The scRNA-Seq expression of the entire glycolytic pathway was analyzed in primary CD4+ T cells infected *in vitro* with VSVG-HIV-1-GFP and sorted for viral expression as detailed in Golumbeanu *et al* (2018). Following latency establishment, cells were left untreated or HIV-1 expression was reactivated through suberoyl anilide hydroxamic acid (SAHA) or α-CD3-CD28 engagement. Clusters 1 (low latency reactivation potential) and 2 (high latency reactivation potential) were identified by principal component analysis as described in Golumbeanu *et al* (2018). T-cell subtypes were identified using the SingleR R package and allocated to each cluster irrespective of the *in vitro* treatment. The expression level of the HUMAN-GLYCOLYSIS pathway was calculated as the average expression of genes comprising the gene list. *n* = 1 donor, 131 cells (Cluster 1), 93 cells (Cluster 2).

© 2021 The Authors

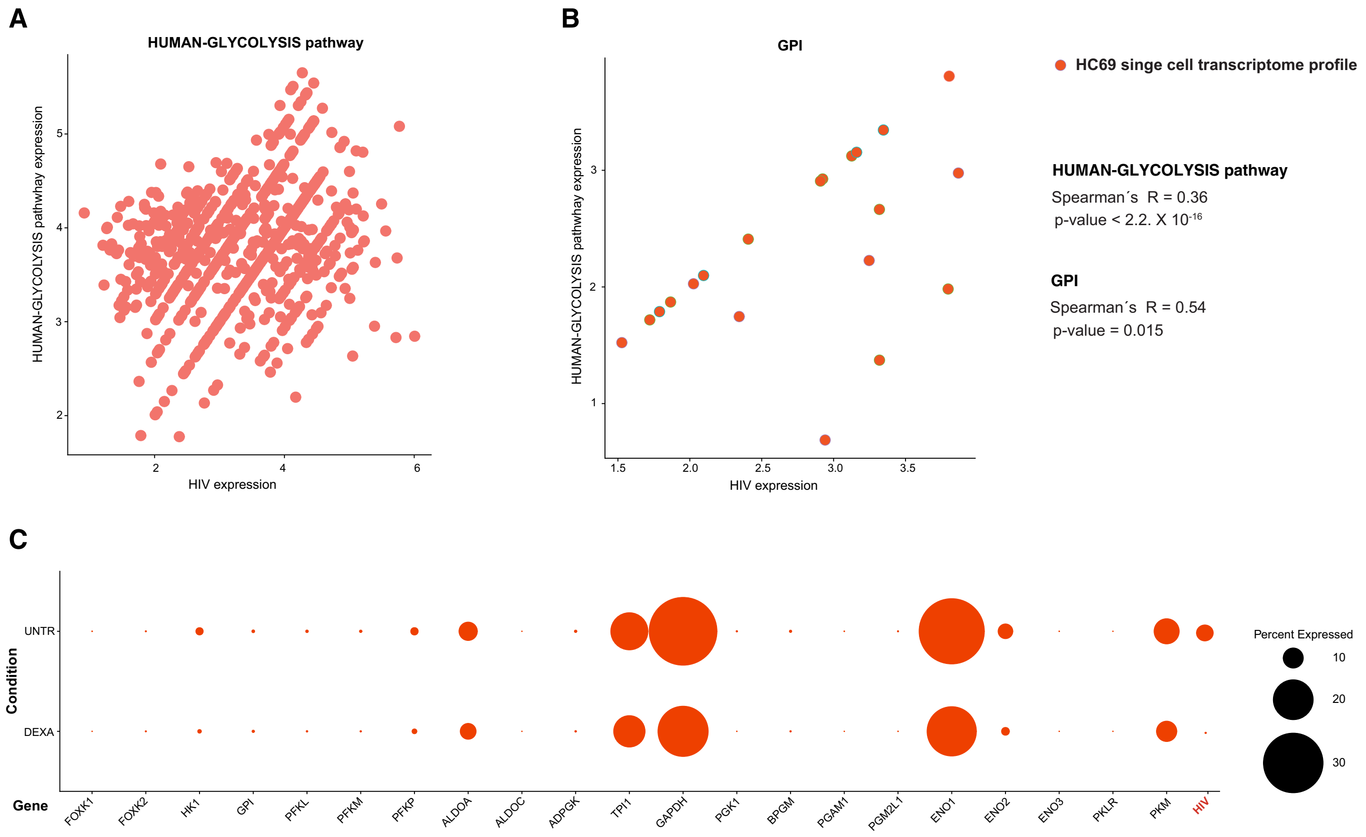

Figure EV2. Single-cell level correlation between glycolytic genes and HIV-1 expression in HC69 microglia.

A, B Correlation of combined HIV-1 expression and the expression of the entire glycolytic pathway (A) or GPI only (B) in scRNA-Seq profiling of untreated HC69 microglial cells. Data were analyzed by Spearman's correlation coefficients.

C Effect of the glycolysis depressing agent dexamethasone (DEXA) on the baseline HIV-1 expression in HC69 microglia cells. HC69 cells were left untreated or treated with DEXA (1 μM) for 72 h and then subjected to scRNA-Seq. The bubble plot depicts the percentage of cells in which the transcriptional expression of HIV-1 or the genes of the HUMAN-GLYCOLYSIS pathway was detectable.

© 2021 The Authors

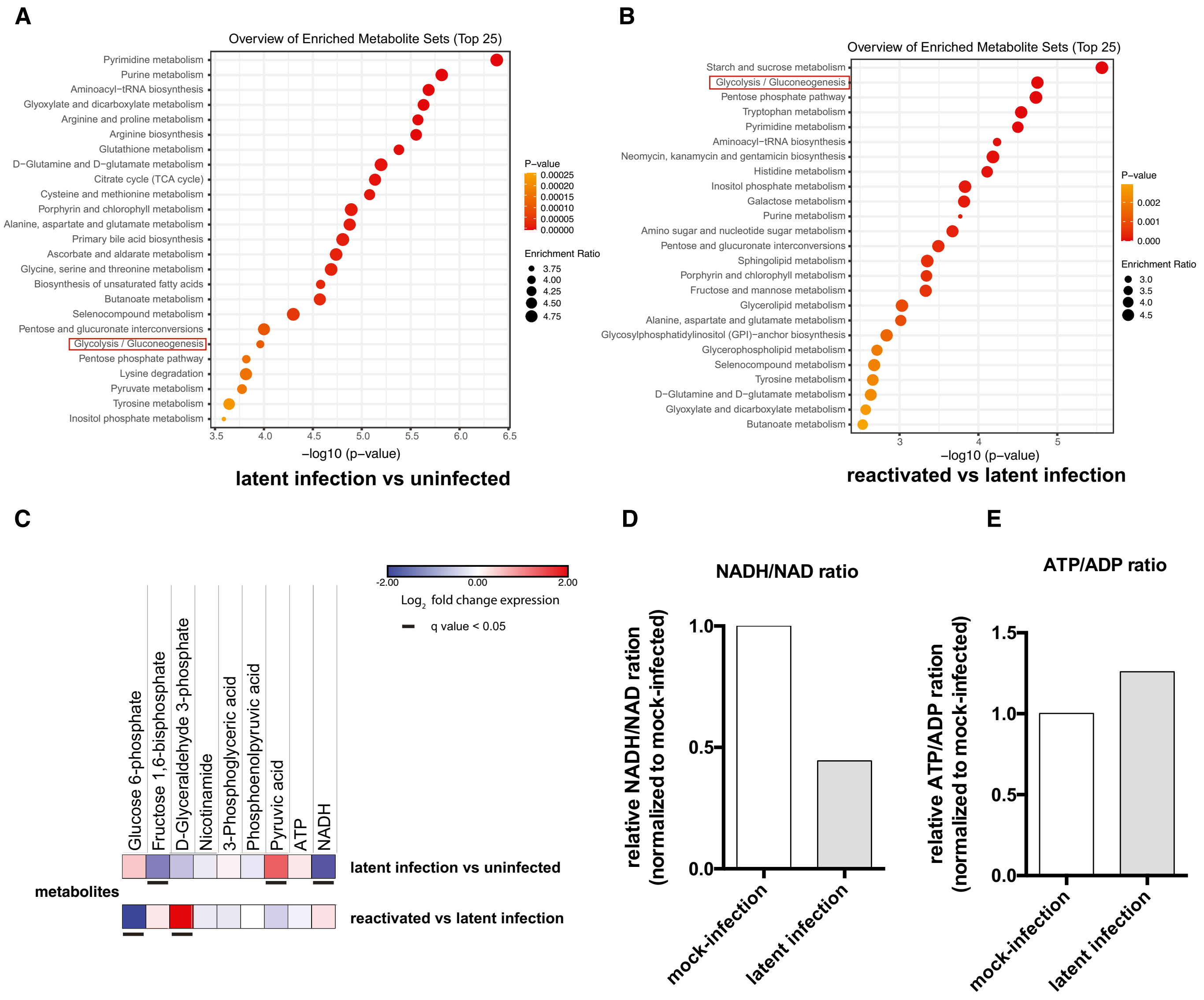

Figure EV3. Modulation of glycolysis and other metabolic pathways during productive or latent HIV-1 infection in microglia cells.

Latently HIV-1-infected (HC69) or uninfected (C20) microglia cells were subjected to metabolomic analysis under unstimulated conditions or following stimulation with TNF to reactivate latent HIV-1.

A, B Metabolite enrichment analysis in latently infected as compared to uninfected cells (A) or in cells reactivated from latency as compared to latently infected cells (B). The top enriched pathways were ordered according to *P*-values obtained by Q statistics for metabolic data sets performed with Globaltest (MetaboAnalyst) (Xia *et al*, 2009).

C Heatmaps of glycolytic metabolites in latently infected HC69 cells as compared to their uninfected counterparts or HC69 cells with HIV-1 reactivated by TNF as compared to latently infected cells. Data are displayed as Log_2 fold change expression. Adjusted *P*-values (*q* values) were calculated by the Benjamini–Hochberg false discovery rate.

D, E Relative ratios of $NADH/NAD^+$ (D) and ATP/ADP (E) in latently infected and reactivated cells. Data were normalized using the matching uninfected control.

© 2021 The Authors

Figure EV4. Schematic depiction of the metabolic networks active during latent HIV-1 infection in microglial cells.

The regulation of metabolic pathways was reconstructed based on the metabolomic data of latently infected microglial cells (HC69) as compared to their uninfected counterpart (C20). Enzymes are shown in italics. The figure includes the main energetic pathways described in the paper, as well as relevant connections with pathways that were significantly enriched in the analysis shown in Fig EV3A. The red lines indicate the pathway proposed to be active in latently infected cells. Solid lines indicate direct connections. Dashed lines indicate indirect connections involving intermediate metabolites not shown in the figure. Networks were built using the Cytoscape software (http://www.cytoscape.org) and the MetScape plugin (http://metscape.ncibi.org/tryplugin.html) (Gao *et al*, 2010) and adapted using Adobe Illustrator (v 16.03). Nicotinamide and glycerolipid pathways were included in the figure as they were found to be significantly enriched [nicotinamide (Q statistic = 45.127, $P = 0.0069702$ and FDR= 0.010205); glycerolipid (Q statistic = 80.501, $P = 0.015301$ and FDR= 0.020291)] in latently infected cells, although they were not comprised in the top 25 list in Fig EV3A. ACLY = ATP citrate lyase; ACO = aconitase; ADPGK = ADP-dependent glucokinase; ALDO = fructose 1,6 bisphosphate aldolase; ENO1 = enolase 1; FBP1 = fructose-bisphosphatase; PFK = phosphofructokinase 1; FH = fumarate hydratase; H6PD = hexose-6-phosphate dehydrogenase; HK1 = hexokinase 1; G6PD = glucose-6-phosphate dehydrogenase; GAPDH = glyceraldehyde 3-phosphate dehydrogenase; GCK = glucokinase; GPDH = glycerol 3-phosphate dehydrogenase; GPI = glucose-6-phosphate isomerase; KORA = ketoglutarate dehydrogenase A; IDH = isocitrate dehydrogenase; LDHAL6A = lactate dehydrogenase A like 6A; MDH1 = malate dehydrogenase 1; PCK1 phosphoenolpyruvate carboxykinase 1; PFK = phosphofructokinase; PGAM4 = phosphoglycerate mutase family member 4; PGD = 6-phosphogluconate dehydrogenase; PGK1 = phosphoglycerate kinase 1; PGLS = 6-phosphogluconolactonase; PGM1 = phosphoglucomutase 1; PKLR = pyruvate kinase L/R; PRPS1 = ribose phosphate pyrophosphokinase 1; RPIA = ribose 5-phosphate isomerase; SDHA = succinate dehydrogenase complex flavoprotein subunit A; SUCD = succinate semialdehyde dehydrogenase; TKT = transketolase; TPI = triose phosphate isomerase.

 © 2021 The Authors

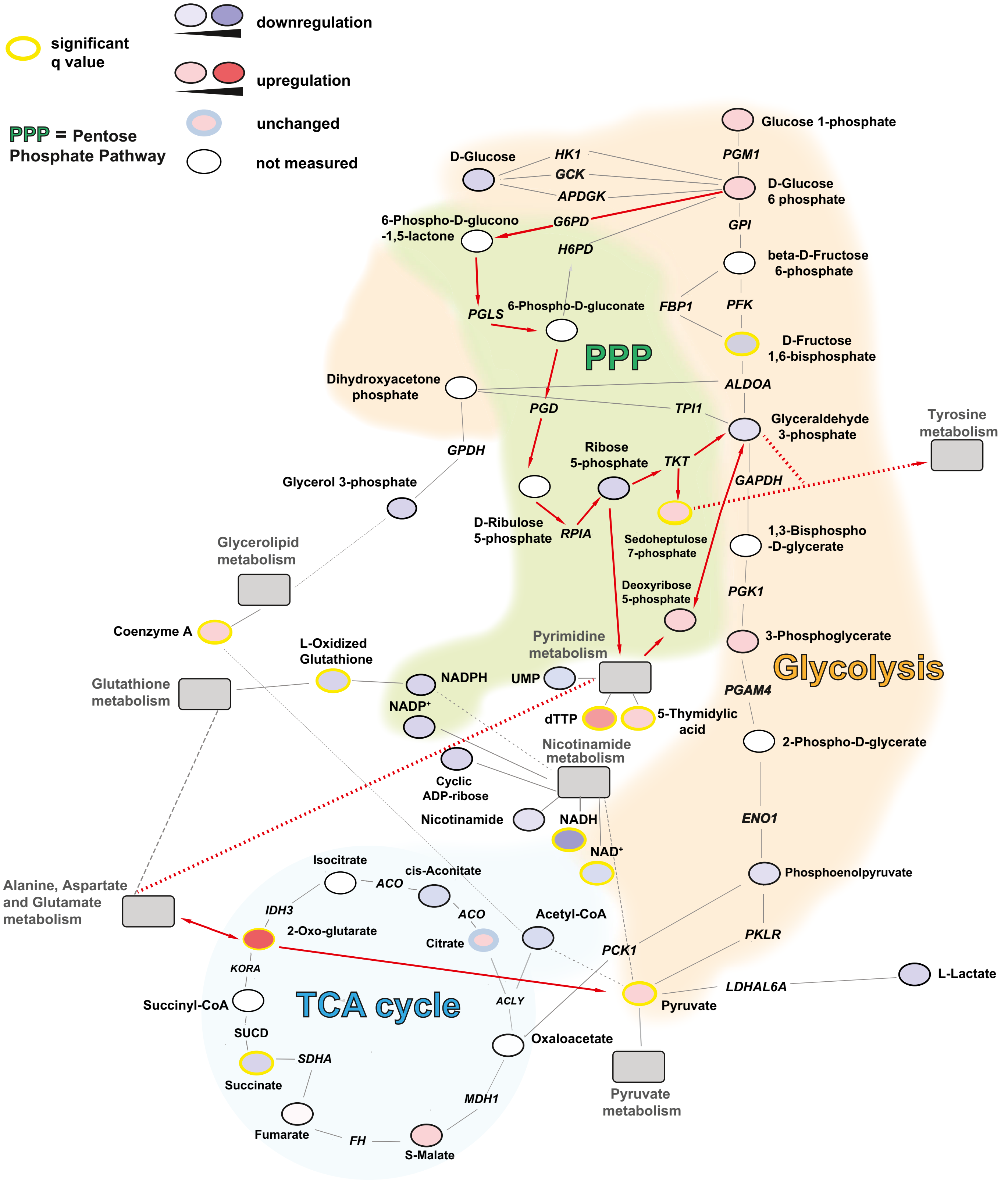
significant
q value
downregulation
upregulation
unchanged
not measured
PPP = Pentose Phosphate Pathway
Glucose 1-phosphate
PGM1
D-Glucose
HK1
GCK
APDGK
D-Glucose 6 phosphate
6-Phospho-D-glucono -1,5-lactone
G6PD
H6PD
GPI
beta-D-Fructose 6-phosphate
PGLS
6-Phospho-D-gluconate
FBP1
PFK
PPP
D-Fructose 1,6-bisphosphate
Dihydroxyacetone phosphate
ALDOA
PGD
TPI1
Glyceraldehyde 3-phosphate
Tyrosine metabolism
GPDH
Ribose 5-phosphate
TKT
GAPDH
Glycerol 3-phosphate
D-Ribulose 5-phosphate
RPIA
Sedoheptulose 7-phosphate
1,3-Bisphospho -D-glycerate
Glycerolipid metabolism
Deoxyribose 5-phosphate
PGK1
Coenzyme A
3-Phosphoglycerate
Pyrimidine metabolism
Glycolysis
L-Oxidized Glutathione
Glutathione metabolism
NADPH
UMP
PGAM4
NADP+
dTTP
5-Thymidylic acid
Nicotinamide metabolism
2-Phospho-D-glycerate
Cyclic ADP-ribose
Nicotinamide
NADH
ENO1
NAD+
Isocitrate
cis-Aconitate
Phosphoenolpyruvate
Alanine, Aspartate and Glutamate metabolism
ACO
IDH3
ACO
Acetyl-CoA
2-Oxo-glutarate
Citrate
PCK1
PKLR
KORA
ACLY
LDHAL6A
L-Lactate
Succinyl-CoA
TCA cycle
Pyruvate
SUCD
Oxaloacetate
SDHA
Succinate
MDH1
Pyruvate metabolism
Fumarate
FH
S-Malate

Figure EV4.

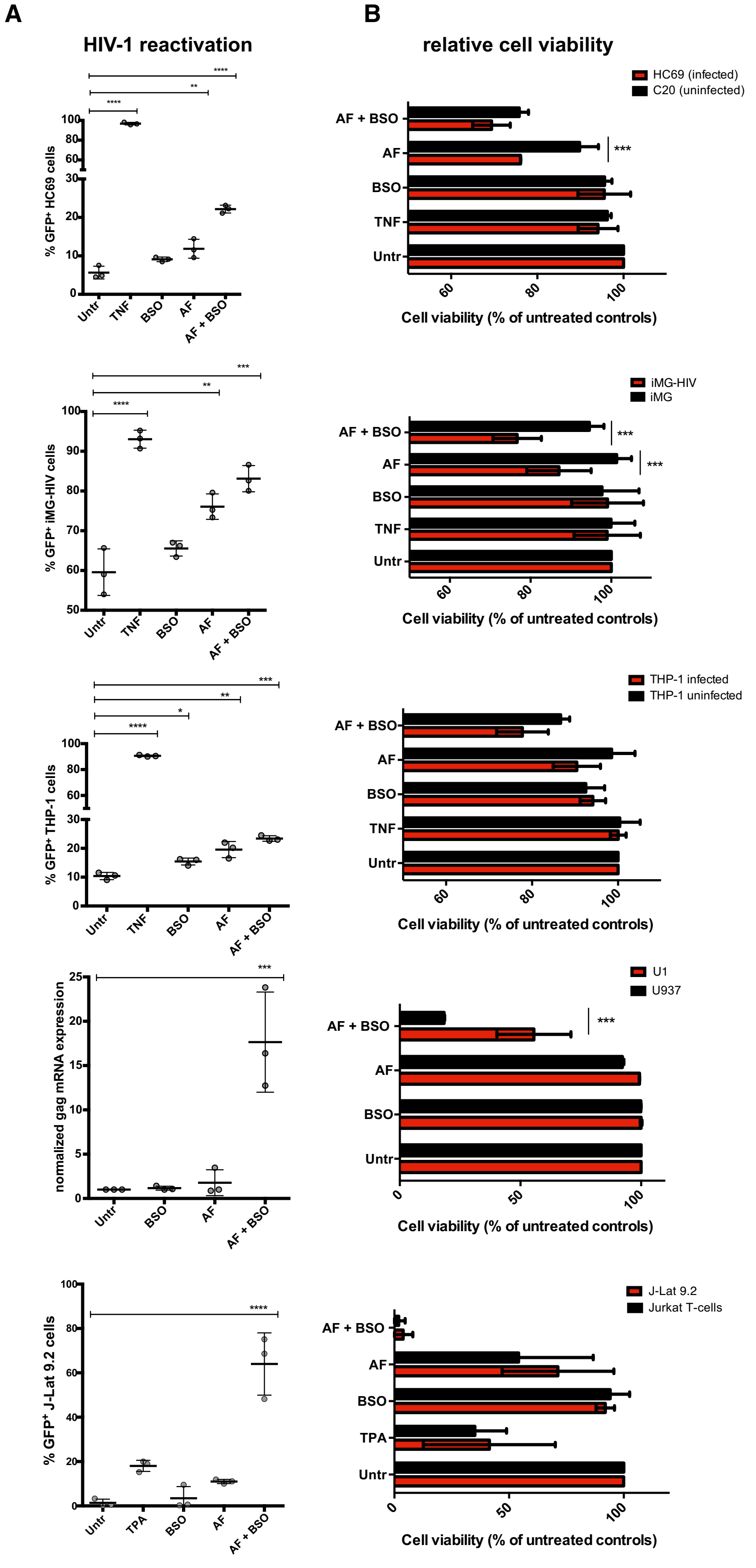

Figure EV5. HIV-1 reactivation (A) and viability (B) in myeloid and lymphoid cells following treatment with auranofin (AF) and buthionine sulfoximine (BSO).

A, B Culturing conditions for each cell type are detailed in the Materials and Methods section. Cells were left untreated or treated with AF (500 nM), BSO (250 µM), or a combination of the two. Treatment duration was 48 h except for Jurkat/J-lat 9.2 and U937/U1 cells that were treated for 24 h. HIV-1 expression was determined by FACS except for U1 cells where it was determined by qPCR. Data are expressed as mean ± SD of three replicates and were analyzed by one-way ANOVA followed by Tukey's post-test (A) or two-way ANOVA followed by Sidak's post-test (B). $^*P < 0.05$, $^{**}P < 0.01$, $^{***}P < 0.001$, $^{****}P < 0.0001$.

 © 2021 The Authors
