# Supplementary material for: Glycolysis downregulation is a hallmark of HIV‐1 latency and sensitizes infected cells to oxidative stress
Source: EMBO Mol Med. 2021 Jul 20;13(8):e13901. doi: 10.15252/emmm.202013901 (PMC8350904; doi:10.15252/emmm.202013901)
Supplement: Supplementary file 2 — Appendix [file EMMM-13-e13901-s002.pdf]

Table of contents

Appendix Table S1. Microarray analysis of the effect of HIV-1 infection on gene expression in $CD4^+$ T-cells.

Appendix Table S2. Characteristics of PLWH enrolled for collection of blood used to assess the *ex-vivo* efficacy of AF and BSO.

Appendix Figure S1. Modulation of glycolytic pathway in the $CD4^+$ T-cell proteome.

Appendix Figure S2. Modulation of glycolytic pathway transcription during productive or latent HIV-1 infection of microglia.

Appendix Figure S3. Single-cell lactate level in HIV-1 infected and uninfected TZM-bl cells.

Appendix Figure S4. Single-cell lipid droplet content in HIV-1 infected and uninfected TZM-bl cells.

Appendix Figure S5. Viro-metabolic effects of exogenous Tat administration in HIV-1 infected and uninfected cells.

Appendix Figure S6. Auranofin (AF) and buthionine sulfoximine (BSO) co-treatment induces synergistic oxidative stress in U1 cells.

Appendix Figure S7. Modulation of glycolytic metabolites in cells of PLWH treated with auranofin (AF) and/or buthionine sulfoximine (BSO).

Appendix Figure S8. Gating strategy and viability of $CD4^+$ T-cells of PLWH under ART left untreated or treated with auranofin (AF) and/or buthionine sulfoximine (BSO).

Appendix Figure S9. HIV-1 reactivation in $CD4^+$ T-cells of PLWH under ART following treatment with auranofin (AF) and buthionine sulfoximine (BSO).

Appendix Table S1. Microarray analysis of the effect of HIV-1 infection on gene expression in $CD4^+$ T-cells. Primary $CD4^+$ T-cells were isolated from total blood of healthy individuals, activated for 72 h with α-CD3-CD28 beads and infected with HIV-1_{pNL4-3} or mock-infected. Both infected and mock-infected cells were cultured for two weeks to model different infection stages as described in (Shytaj *et al*, 2020). On days 7, 9 and 14 post-infection, HIV-1-infected and mock-infected cells were subjected to microarray analysis. Data from different time points were pooled for the analysis. Significantly enriched pathways in HIV-1 infected and mock-infected cells were identified by Gene Set Enrichment Analysis [GSEA (Subramanian *et al*, 2005)]. Number of donors = 2.

Reactome

Activated in infected

Gene set	NES	FDR q-value
CELL CYCLE	8.91	0
CELL CYCLE MITOTIC	8.58	0
DNA REPLICATION	8.45	0
IMMUNE SYSTEM	8.04	0
MITOTIC M M G1 PHASES	7.70	0
HIV INFECTION	7.15	0
S PHASE	7.01	0
SYNTHESIS OF DNA	6.99	0
MITOTIC G1 G1 S PHASES	6.84	0
ADAPTIVE IMMUNE SYSTEM	6.76	0
MRNA PROCESSING	6.71	0
G1 S TRANSITION	6.56	0
HOST INTERACTIONS OF HIV FACTORS	6.49	0
ORC1 REMOVAL FROM CHROMATIN	6.44	0
ASSEMBLY OF THE PRE REPLICATIVE COMPLEX	6.39	0
M G1 TRANSITION	6.39	0
PROCESSING OF CAPPED INTRON CONTAINING PRE MRNA	6.31	0
TRANSCRIPTION	6.16	0
METABOLISM OF RNA	5.87	0
INTERFERON SIGNALING	5.79	0
SIGNALING BY THE B CELL RECEPTOR BCR	5.77	0
VIF MEDIATED DEGRADATION OF APOBEC3G	5.67	0
RNA POL II TRANSCRIPTION	5.65	0
CYTOKINE SIGNALING IN IMMUNE SYSTEM	5.64	0
SCFSKP2 MEDIATED DEGRADATION OF P27 P21	5.62	0
CDT1 ASSOCIATION WITH THE CDC6 ORC ORIGIN COMPLEX	5.59	0
CYCLIN E ASSOCIATED EVENTS DURING G1 S TRANSITION	5.57	0
CELL CYCLE CHECKPOINTS	5.55	0
MRNA SPLICING	5.53	0
CDK MEDIATED PHOSPHORYLATION AND REMOVAL OF CDC6	5.53	0
REGULATION OF MRNA STABILITY BY PROTEINS THAT BIND AU RICH ELEMENTS	5.51	0
ER PHAGOSOME PATHWAY	5.44	0
REGULATION OF ORNITHINE DECARBOXYLASE ODC	5.42	0
P53 DEPENDENT G1 DNA DAMAGE RESPONSE	5.42	0
LATE PHASE OF HIV LIFE CYCLE	5.41	0
DESTABILIZATION OF MRNA BY AUF1 HNRNP D0	5.38	0
AUTODEGRADATION OF THE E3 UBIQUITIN LIGASE COP1	5.29	0
P53 INDEPENDENT G1 S DNA DAMAGE CHECKPOINT	5.29	0
SCF BETA TRCP MEDIATED DEGRADATION OF EMI1	5.26	0
MITOTIC PROMETAPHASE	5.20	0
ACTIVATION OF NF KAPPAB IN B CELLS	5.20	0
HIV LIFE CYCLE	5.17	0
DOWNSTREAM SIGNALING EVENTS OF B CELL RECEPTOR BCR	5.10	0
REGULATION OF MITOTIC CELL CYCLE	5.07	0
CROSS PRESENTATION OF SOLUBLE EXOGENOUS ANTIGENS ENDOSOMES	5.02	0
ANTIVIRAL MECHANISM BY IFN STIMULATED GENES	4.98	0
APOPTOSIS	4.94	0
ANTIGEN PROCESSING CROSS PRESENTATION	4.93	0
APC C CDH1 MEDIATED DEGRADATION OF CDC20 AND OTHER APC C CDH1 TARGETED PROTEINS IN LATE MITOSIS EARLY G1	4.88	0
REGULATION OF APOPTOSIS	4.84	0
SIGNALING BY WNT	4.82	0
METABOLISM OF PROTEINS	4.80	0
CLASS I MHC MEDIATED ANTIGEN PROCESSING PRESENTATION	4.79	0
AUTODEGRADATION OF CDH1 BY CDH1 APC C	4.70	0
APC C CDC20 MEDIATED DEGRADATION OF MITOTIC PROTEINS	4.65	0
METABOLISM OF MRNA	4.62	0
ANTIGEN PROCESSING UBIQUITINATION PROTEASOME DEGRADATION	4.61	0
TRANSPORT OF MATURE TRANSCRIPT TO CYTOPLASM	4.59	0
RNA POL I RNA POL III AND MITOCHONDRIAL TRANSCRIPTION	4.36	0
FORMATION OF RNA POL II ELONGATION COMPLEX	4.33	0
METABOLISM OF NON CODING RNA	4.30	0
DNA REPAIR	4.20	0
UNFOLDED PROTEIN RESPONSE	4.19	0
TRANSPORT OF MATURE MRNA DERIVED FROM AN INTRONLESS TRANSCRIPT	4.15	0
RNA POL II PRE TRANSCRIPTION EVENTS	4.15	0
CLEAVAGE OF GROWING TRANSCRIPT IN THE TERMINATION REGION	4.09	0
FORMATION OF THE HIV1 EARLY ELONGATION COMPLEX	4.02	0
CHROMOSOME MAINTENANCE	4.02	0
MITOTIC G2 G2 M PHASES	3.99	0
TCA CYCLE AND RESPIRATORY ELECTRON TRANSPORT	3.94	0
CHEMIOSMOTIC COUPLING AND HEAT PRODUCTION BY UNCOUPLING PROTEINS	3.89	0
RESPIRATORY ELECTRON TRANSPORT	3.85	0
METABOLISM OF AMINO ACIDS AND DERIVATIVES	3.82	0
DIABETES PATHWAYS	3.82	0
DNA STRAND ELONGATION	3.77	0
MITOCHONDRIAL PROTEIN IMPORT	3.75	0
MRNA CAPPING	3.73	0

Activated in Mock

Gene set	NES	FDR q-value
3 UTR MEDIATED TRANSLATIONAL REGULATION	-5.75	0
PEPTIDE CHAIN ELONGATION	-5.54	0
TRANSLATION	-5.40	0
SRP DEPENDENT COTRANSLATIONAL PROTEIN TARGETING TO MEMBRANE	-4.97	0
NONSENSE MEDIATED DECAY ENHANCED BY THE EXON JUNCTION COMPLEX	-4.84	0
INFLUENZA VIRAL RNA TRANSCRIPTION AND REPLICATION	-4.76	0
INFLUENZA LIFE CYCLE	-3.79	0
ACTIVATION OF THE MRNA UPON BINDING OF THE CAP BINDING COMPLEX AND EIFS AND SUBSEQUENT BINDING TO 43S	-3.78	0
FORMATION OF THE TERNARY COMPLEX AND SUBSEQUENTLY THE 43S COMPLEX	-3.54	0
OLFACTORY SIGNALING PATHWAY	-3.19	0
GENERIC TRANSCRIPTION PATHWAY	-3.17	0
GPCR DOWNSTREAM SIGNALING	-2.72	0.0006
TCR SIGNALING	-2.68	0.0007
GENERATION OF SECOND MESSENGER MOLECULES	-2.46	0.0033
GLYCOLYSIS	-2.38	0.0055
BINDING AND ENTRY OF HIV VIRION	-2.14	0.0266
PROTEOLYTIC CLEAVAGE OF SNARE COMPLEX PROTEINS	-2.06	0.0440
HS GAG DEGRADATION	-2.05	0.0445

Biocarta

Activated in infected

Gene set	NES	FDR q-value
PROTEASOME PATHWAY	4.67	0
CELLCYCLE PATHWAY	3.39	0
CASPASE PATHWAY	3.16	0
MCM PATHWAY	2.92	0
HIVNEF PATHWAY	2.72	0.0005
FAS PATHWAY	2.51	0.0018
RANMS PATHWAY	2.53	0.0019
ATRBRCA PATHWAY	2.54	0.0022
FCER1 PATHWAY	2.58	0.0023
BCELLSURVIVAL PATHWAY	2.34	0.0063
G1 PATHWAY	2.35	0.0065
PGC1A PATHWAY	2.37	0.0068
MITOCHONDRIA PATHWAY	2.35	0.0071
RACCYCD PATHWAY	2.37	0.0073
MAPK PATHWAY	2.30	0.0076
G2 PATHWAY	2.27	0.0080
DNAFRAGMENT PATHWAY	2.27	0.0084
CHEMICAL PATHWAY	2.27	0.0088
BCR PATHWAY	2.27	0.0092
HDAC PATHWAY	2.22	0.0096
EIF2 PATHWAY	2.22	0.0099
NDKDYNAMIN PATHWAY	2.17	0.0102
RHO PATHWAY	2.19	0.0102
MPR PATHWAY	2.18	0.0105
MEF2D PATHWAY	2.17	0.0105
SRCRPTP PATHWAY	2.18	0.0106
DEATH PATHWAY	2.13	0.0114
SET PATHWAY	2.12	0.0116
SKP2E2F PATHWAY	2.08	0.0148
TNFR1 PATHWAY	2.06	0.0166
P35ALZHEIMERS PATHWAY	2.03	0.0188
PTDINS PATHWAY	2.02	0.0193
RNA PATHWAY	2.01	0.0199
D4GDI PATHWAY	1.97	0.0245
P27 PATHWAY	1.95	0.0270
RB PATHWAY	1.94	0.0272
ERK5 PATHWAY	1.93	0.0273
PTC1 PATHWAY	1.95	0.0275
MTOR PATHWAY	1.94	0.0275
P53HYPOXIA PATHWAY	1.93	0.0278
VDR PATHWAY	1.87	0.0356
AKAP95 PATHWAY	1.87	0.0358
CYTOKINE PATHWAY	1.87	0.0364
MCALPAIN PATHWAY	1.86	0.0367
NFAT PATHWAY	1.86	0.0368
ETC PATHWAY	1.85	0.0371
GCR PATHWAY	1.84	0.0390
CARM ER PATHWAY	1.82	0.0424
FMLP PATHWAY	1.81	0.0434
EIF PATHWAY	1.81	0.0435
IL3 PATHWAY	1.81	0.0437
BLYMPHOCYTE PATHWAY	1.79	0.0472

Activated in Mock

Gene set	NES	FDR q-value
IL10 PATHWAY	-2.76	0.0014
PDGF PATHWAY	-2.36	0.0116
EGF PATHWAY	-2.36	0.0174
TCRA PATHWAY	-2.15	0.0323
RELA PATHWAY	-2.05	0.0336
IL7 PATHWAY	-2.09	0.0347
IL2 PATHWAY	-2.10	0.0365
TCAPOPTOSIS PATHWAY	-2.05	0.0365
TCR PATHWAY	-2.15	0.0402
GLYCOLYSIS PATHWAY	-1.98	0.0457

NEP NS2 INTERACTS WITH THE CELLULAR EXPORT MACHINERY	3.66	0
TRANSPORT OF RIBONUCLEOPROTEINS INTO THE HOST NUCLEUS	3.65	0
INTERFERON ALPHA BETA SIGNALING	3.64	0
INTERACTIONS OF VPR WITH HOST CELLULAR PROTEINS	3.60	0
MRNA SPLICING MINOR PATHWAY	3.54	0
TRNA AMINOACYLATION	3.53	0
TRANSCRIPTION COUPLED NER TC NER	3.45	0
MHC CLASS II ANTIGEN PRESENTATION	3.44	0
MRNA 3 END PROCESSING	3.43	0
PROTEIN FOLDING	3.43	0
RECRUITMENT OF MITOTIC CENTROSOME PROTEINS AND COMPLEXES	3.41	0
ELONGATION ARREST AND RECOVERY	3.40	0
PROCESSING OF CAPPED INTRONLESS PRE MRNA	3.40	0
MICRORNA MIRNA BIOGENESIS	3.38	0
ACTIVATION OF THE PRE REPLICATIVE COMPLEX	3.36	0
CHOLESTEROL BIOSYNTHESIS	3.32	0
SIGNALLING BY NGF	3.30	0
ABORTIVE ELONGATION OF HIV1 TRANSCRIPT IN THE ABSENCE OF TAT	3.27	0
RNA POL II TRANSCRIPTION PRE INITIATION AND PROMOTER OPENING	3.27	0
RNA POL III TRANSCRIPTION	3.25	0
NUCLEOTIDE EXCISION REPAIR	3.23	0
ACTIVATION OF CHAPERONE GENES BY XBP1S	3.23	0
EXTENSION OF TELOMERES	3.22	0
INTRINSIC PATHWAY FOR APOPTOSIS	3.22	0
RNA POL III TRANSCRIPTION INITIATION FROM TYPE 3 PROMOTER	3.21	0
RNA POL I TRANSCRIPTION	3.21	0
RNA POL III TRANSCRIPTION INITIATION FROM TYPE 2 PROMOTER	3.18	0
PREFOLDIN MEDIATED TRANSFER OF SUBSTRATE TO CCT TRIC	3.16	0
ACTIVATION OF ATR IN RESPONSE TO REPLICATION STRESS	3.15	0
REGULATION OF GLUCOKINASE BY GLUCOKINASE REGULATORY PROTEIN	3.14	0
LOSS OF NLP FROM MITOTIC CENTROSOMES	3.14	0
TELOMERE MAINTENANCE	3.12	0
REGULATORY RNA PATHWAYS	3.09	0
CYTOSOLIC TRNA AMINOACYLATION	3.08	0
G0 AND EARLY G1	3.06	0
E2F MEDIATED REGULATION OF DNA REPLICATION	3.04	0
SIGNALING BY EGFR IN CANCER	3.03	0
MEIOSIS	3.01	0
RNA POL III TRANSCRIPTION TERMINATION	3.00	0
RNA POL III CHAIN ELONGATION	2.99	0
UNWINDING OF DNA	2.90	2.24E-05
ASSOCIATION OF TRIC CCT WITH TARGET PROTEINS DURING BIOSYNTHESIS	2.92	2.26E-05
G2 M CHECKPOINTS	2.95	2.28E-05
ANTIGEN ACTIVATES B CELL RECEPTOR LEADING TO GENERATION OF SECOND MESSENGERS	2.83	3.24E-05
INTERFERON GAMMA SIGNALING	2.86	3.27E-05
METABOLISM OF LIPIDS AND LIPOPROTEINS	2.88	3.30E-05
AMINO ACID SYNTHESIS AND INTERCONVERSION TRANSAMINATION	2.88	3.32E-05
MEMBRANE TRAFFICKING	2.88	3.35E-05
FORMATION OF TUBULIN FOLDING INTERMEDIATES BY CCT TRIC	2.81	5.46E-05
RNA POL I TRANSCRIPTION TERMINATION	2.77	6.54E-05
DEPOSITION OF NEW CENPA CONTAINING NUCLEOSOMES AT THE CENTROMERE	2.76	8.69E-05
MEIOTIC RECOMBINATION	2.71	1.07E-04
PROCESSING OF INTRONLESS PRE MRNAS	2.73	1.08E-04
ASPARAGINE N LINKED GLYCOSYLATION	2.68	1.14E-04
NGF SIGNALLING VIA TRKA FROM THE PLASMA MEMBRANE	2.70	1.15E-04
IMMUNOREGULATORY INTERACTIONS BETWEEN A LYMPHOID AND A NON LYMPHOID CELL	2.70	1.16E-04
RNA POL I TRANSCRIPTION INITIATION	2.70	1.17E-04
G1 PHASE	2.68	1.23E-04
FORMATION OF TRANSCRIPTION COUPLED NER TC NER REPAIR COMPLEX	2.67	1.33E-04
GLOBAL GENOMIC NER GG NER	2.65	1.62E-04
GLUCOSE TRANSPORT	2.64	2.01E-04
LAGGING STRAND SYNTHESIS	2.62	2.67E-04
REPAIR SYNTHESIS FOR GAP FILLING BY DNA POL IN TC NER	2.62	2.75E-04
VITAMIN B5 PANTOTHENATE METABOLISM	2.61	2.84E-04
G1 S SPECIFIC TRANSCRIPTION	2.56	3.70E-04
VIRAL MESSENGER RNA SYNTHESIS	2.53	4.34E-04
LINKED OLIGOSACCHARIDE LLO AND TRANSFER TO A NASCENT PROTEIN	2.53	4.47E-04
PERK REGULATED GENE EXPRESSION	2.53	4.50E-04
ACTIVATION OF BH3 ONLY PROTEINS	2.50	5.40E-04
SIGNALING BY SCF KIT	2.50	5.64E-04
DESTABILIZATION OF MRNA BY KSRP	2.49	5.78E-04
SLBP DEPENDENT PROCESSING OF REPLICATION DEPENDENT HISTONE PRE MRNAS	2.49	5.82E-04
SIGNALING BY ILS	2.48	6.14E-04
TOLL RECEPTOR CASCADES	2.47	6.64E-04
NUCLEOTIDE BINDING DOMAIN LEUCINE RICH REPEAT CONTAINING RECEPTOR NLR SIGNALING PATHWAYS	2.46	6.74E-04
SIGNALING BY ERBB2	2.46	6.79E-04
MRNA DECAY BY 3 TO 5 EXORIBONUCLEASE	2.45	7.06E-04
TRAF3 DEPENDENT IRF ACTIVATION PATHWAY	2.45	7.10E-04
CYCLIN A B1 ASSOCIATED EVENTS DURING G2 M TRANSITION	2.44	7.49E-04
POL SWITCHING	2.43	8.51E-04

FACTORS INVOLVED IN MEGAKARYOCYTE DEVELOPMENT AND PLATELET PRODUCTION	2.42	9.34E-04
TRIGLYCERIDE BIOSYNTHESIS	2.40	0.0010
POST TRANSLATIONAL PROTEIN MODIFICATION	2.37	0.0014
METABOLISM OF CARBOHYDRATES	2.36	0.0014
NEGATIVE REGULATORS OF RIG I MDA5 SIGNALING	2.36	0.0014
DEADENYLATION DEPENDENT MRNA DECAY	2.34	0.0016
HEMOSTASIS	2.30	0.0021
DARPP 32 EVENTS	2.30	0.0021
MITOCHONDRIAL TRNA AMINOACYLATION	2.30	0.0021
FATTY ACID TRIACYLGLYCEROL AND KETONE BODY METABOLISM	2.29	0.0022
DOUBLE STRAND BREAK REPAIR	2.29	0.0022
RECYCLING PATHWAY OF L1	2.29	0.0022
CITRIC ACID CYCLE TCA CYCLE	2.28	0.0023
PURINE RIBONUCLEOSIDE MONOPHOSPHATE BIOSYNTHESIS	2.28	0.0024
MEIOTIC SYNAPSIS	2.26	0.0027
GAB1 SIGNALOSOME	2.24	0.0030
INNATE IMMUNE SYSTEM	2.25	0.0030
NRAGE SIGNALS DEATH THROUGH JNK	2.23	0.0034
ACTIVATION OF GENES BY ATF4	2.22	0.0036
DOWNSTREAM SIGNAL TRANSDUCTION	2.22	0.0036
REMOVAL OF THE FLAP INTERMEDIATE FROM THE C STRAND	2.17	0.0048
DESTABILIZATION OF MRNA BY TRISTETRAPROLIN TTP	2.17	0.0050
TRAF6 MEDIATED IRF7 ACTIVATION IN TLR7 8 OR 9 SIGNALING	2.16	0.0050
RNA POL I PROMOTER OPENING	2.14	0.0056
P75 NTR RECEPTOR MEDIATED SIGNALLING	2.14	0.0057
SIGNALING BY FGFR	2.13	0.0060
PROCESSIVE SYNTHESIS ON THE LAGGING STRAND	2.12	0.0062
SHC1 EVENTS IN EGFR SIGNALING	2.12	0.0063
CD28 CO STIMULATION	2.12	0.0066
NOD1 2 SIGNALING PATHWAY	2.10	0.0072
TRIF MEDIATED TLR3 SIGNALING	2.10	0.0073
DESTABILIZATION OF MRNA BY BRF1	2.10	0.0074
FANCONI ANEMIA PATHWAY	2.09	0.0077
TRANS GOLGI NETWORK VESICLE BUDDING	2.04	0.0101
OPIOID SIGNALLING	2.04	0.0106
COSTIMULATION BY THE CD28 FAMILY	2.03	0.0112
RIG I MDA5 MEDIATED INDUCTION OF IFN ALPHA BETA PATHWAYS	2.02	0.0115
NFKB ACTIVATION THROUGH FADD RIP1 PATHWAY MEDIATED BY CASPASE 8 AND10	2.00	0.0127
IL 2 SIGNALING	1.99	0.0139
ACTIVATED TLR4 SIGNALLING	1.98	0.0143
ACTIVATION OF CHAPERONES BY ATF6 ALPHA	1.98	0.0143
PRE NOTCH EXPRESSION AND PROCESSING	1.97	0.0147
SIGNALING BY FGFR IN DISEASE	1.97	0.0150
METABOLISM OF NUCLEOTIDES	1.96	0.0160
PI METABOLISM	1.95	0.0162
CHEMOKINE RECEPTORS BIND CHEMOKINES	1.95	0.0162
CDC6 ASSOCIATION WITH THE ORC ORIGIN COMPLEX	1.95	0.0166
IL RECEPTOR SHC SIGNALING	1.94	0.0172
NEF MEDIATES DOWN MODULATION OF CELL SURFACE RECEPTORS BY RECRUITING THEM TO CLATHRIN ADAPTERS	1.93	0.0175
LYSOSOME VESICLE BIOGENESIS	1.93	0.0184
SIGNALLING TO RAS	1.92	0.0194
SOS MEDIATED SIGNALLING	1.91	0.0204
SIGNALING BY GPCR	1.90	0.0207
RESOLUTION OF AP SITES VIA THE MULTIPLE NUCLEOTIDE PATCH REPLACEMENT PATHWAY	1.90	0.0210
APOPTOSIS INDUCED DNA FRAGMENTATION	1.90	0.0212
PIP3 ACTIVATES AKT SIGNALING	1.89	0.0213
GOLGI ASSOCIATED VESICLE BIOGENESIS	1.89	0.0213
SLC MEDIATED TRANSMEMBRANE TRANSPORT	1.89	0.0216
IL 3 5 AND GM CSF SIGNALING	1.89	0.0217
TRANSPORT OF INORGANIC CATIONS ANIONS AND AMINO ACIDS OLIGOPEPTIDES	1.88	0.0227
SHC MEDIATED SIGNALLING	1.88	0.0228
CELL DEATH SIGNALLING VIA NRAGE NRIF AND NADE	1.87	0.0240
DOWNSTREAM SIGNALING OF ACTIVATED FGFR	1.87	0.0243
SYNTHESIS OF PA	1.86	0.0245
THE NLRP3 INFLAMMASOME	1.84	0.0267
TRAF6 MEDIATED INDUCTION OF NFKB AND MAP KINASES UPON TLR7 8 OR 9 ACTIVATION	1.84	0.0272
ENDOSOMAL SORTING COMPLEX REQUIRED FOR TRANSPORT ESCRT	1.82	0.0295
ACETYLCHOLINE BINDING AND DOWNSTREAM EVENTS	1.81	0.0312
SIGNALING BY PDGF	1.81	0.0325
MRNA DECAY BY 5 TO 3 EXORIBONUCLEASE	1.80	0.0336
SIGNALING BY ERBB4	1.79	0.0344
TRAF6 MEDIATED NFKB ACTIVATION	1.79	0.0345
PHOSPHOLIPID METABOLISM	1.79	0.0345
PEROXISOMAL LIPID METABOLISM	1.79	0.0345
DEVELOPMENTAL BIOLOGY	1.79	0.0346
EGFR DOWNREGULATION	1.78	0.0358
GAP JUNCTION TRAFFICKING	1.78	0.0362
PACKAGING OF TELOMERE ENDS	1.77	0.0373
GLUCURONIDATION	1.77	0.0383
G ALPHA1213 SIGNALLING EVENTS	1.76	0.0392

P38MAPK EVENTS	1.76	0.0400
HOMOLOGOUS RECOMBINATION REPAIR OF REPLICATION INDEPENDENT DOUBLE STRAND BREAKS	1.76	0.0402
ASSOCIATION OF LICENSING FACTORS WITH THE PRE REPLICATIVE COMPLEX	1.75	0.0413
TETRAHYDROBIOPTERIN BH4 SYNTHESIS RECYCLING SALVAGE AND REGULATION	1.75	0.0414
CELL CELL JUNCTION ORGANIZATION	1.75	0.0417
INTEGRIN CELL SURFACE INTERACTIONS	1.74	0.0417
SPRY REGULATION OF FGF SIGNALING	1.74	0.0422
FORMATION OF INCISION COMPLEX IN GG NER	1.74	0.0424
INTEGRIN ALPHAIIB BETA3 SIGNALING	1.74	0.0426
FATTY ACYL COA BIOSYNTHESIS	1.74	0.0427
SHC1 EVENTS IN ERBB4 SIGNALING	1.73	0.0430
AMYLOIDS	1.72	0.0450
TRANSMEMBRANE TRANSPORT OF SMALL MOLECULES	1.72	0.0451
ENOS ACTIVATION AND REGULATION	1.72	0.0452
EXTRACELLULAR MATRIX ORGANIZATION	1.72	0.0453
MAP KINASE ACTIVATION IN TLR CASCADE	1.72	0.0458
SYNTHESIS OF SUBSTRATES IN N GLYCAN BIOSYTHESIS	1.71	0.0480
SIGNALLING TO ERKS	1.70	0.0492
COPI MEDIATED TRANSPORT	1.70	0.0492

Appendix Table S1

Appendix Table S2. Characteristics of PLWH enrolled for collection of blood used to assess the *ex-vivo* efficacy of AF and BSO.

Patient ID	Age at sampling	Sex	Stage in which treatment was initiated	Number of years from diagnosis until sampling	Type of ART at initiation (NRTi + protease inhibitor or NNRTi)	CD4 count	Viremia	CD4/CD8 ratio
**donor 1**	52.2	F	A2, Treated undetectable	24.41	AZT	657	<20	1.07
**donor 2**	41.1	M	A3, Treated undetectable	15.88	Retrovir - Invirase - Viracept	323	<20	1.03
**donor 3**	34	F	B1, Treated undetectable	0.18	Dolutégravir/Abacavir/Lamivudine	972	<20	0.97
**donor 4**	38	M	C3, Treated undetectable	5.7	Efavirenz/Emtricitabine/Tenofovir	235	<20	0.209
**donor 5**	58.3	M	B3, Treated undetectable	13.35	AZT-3TC-EFV	664	22	0.68
**donor 6**	43.6	F	B2, Treated undetectable	11.85	Lamivudin + Lopinavir/Ritonavir + Abacavir	1114	<20	1.37
**donor 7**	49.5	M	A3, Treated undetectable	9.16	AZT + 3TC + EFV	543	<20	0.71
**donor 8**	27.7	F	C3, Treated undetectable	8.03	Emtricitabine/tenofovir, Nevirapine	481	<20	1.1

Appendix Table S2

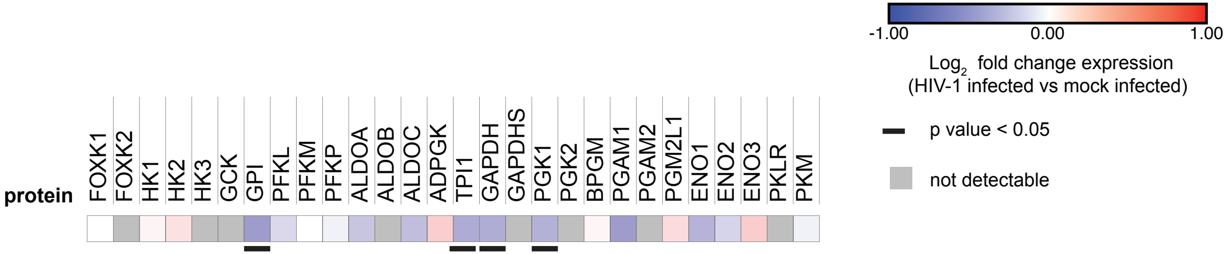

Appendix Figure S1

Appendix Figure S1. Modulation of glycolytic pathway in the CD4$^+$ T-cell proteome. Heatmap of the expression of the glycolytic pathway in HIV-1 infected vs mock-infected cells. Cells were activated for 72 h with α-CD3-CD28 beads and infected with HIV-1$_{pNL4-3}$ or mock-infected. Both infected and mock-infected cells were cultured for two weeks to model different infection stages as described in (Shytaj *et al*, 2020). On days 3, 7, 9 and 14 post-infection, HIV-1 infected and mock-infected cells were subjected to proteomic analysis (Shytaj *et al*, 2020). Data were analyzed by Student's t-test after pooling the different time points.

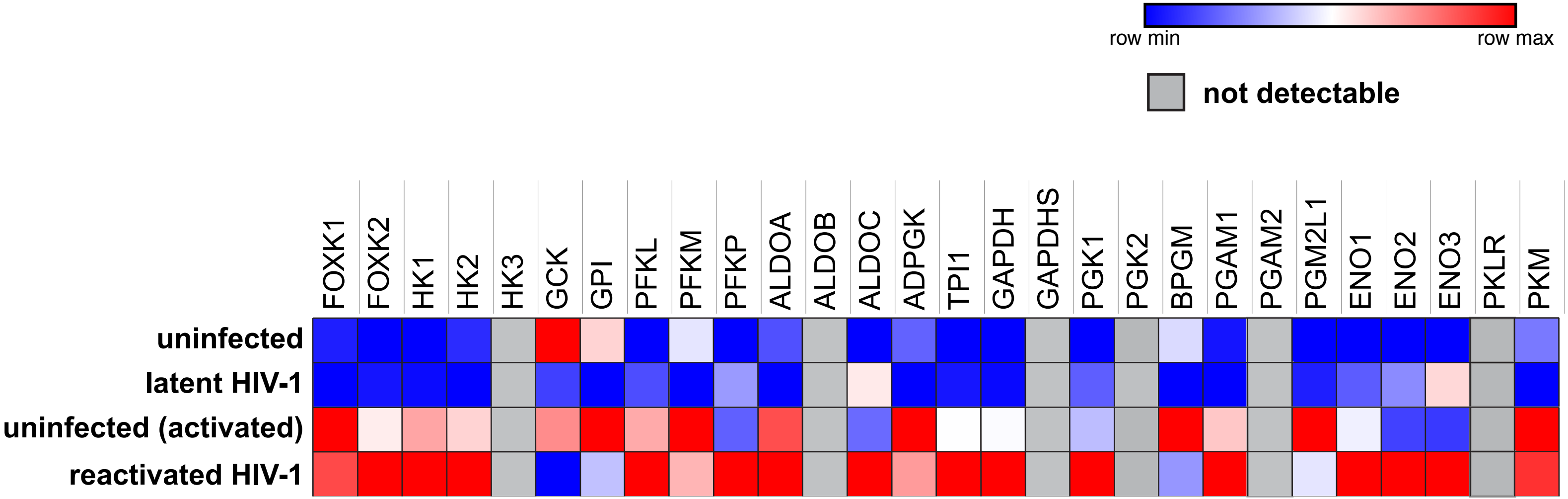

Appendix Figure S2

Appendix Figure S2. **Modulation of glycolytic pathway transcription during productive or latent HIV-1 infection of microglia.** Heatmap of the relative gene expression of the glycolytic pathway in C20/HC69 microglial cells (Garcia-Mesa *et al*, 2017) is shown. Microglial cells were subjected to RNA-Seq analysis under four conditions: uninfected (C20), uninfected and activated (C20-TNF), latently HIV-1 infected (HC69) and reactivated HIV-1 infection (HC69-TNF). Expression data were analyzed by Deseq2 (Love *et al*, 2014).

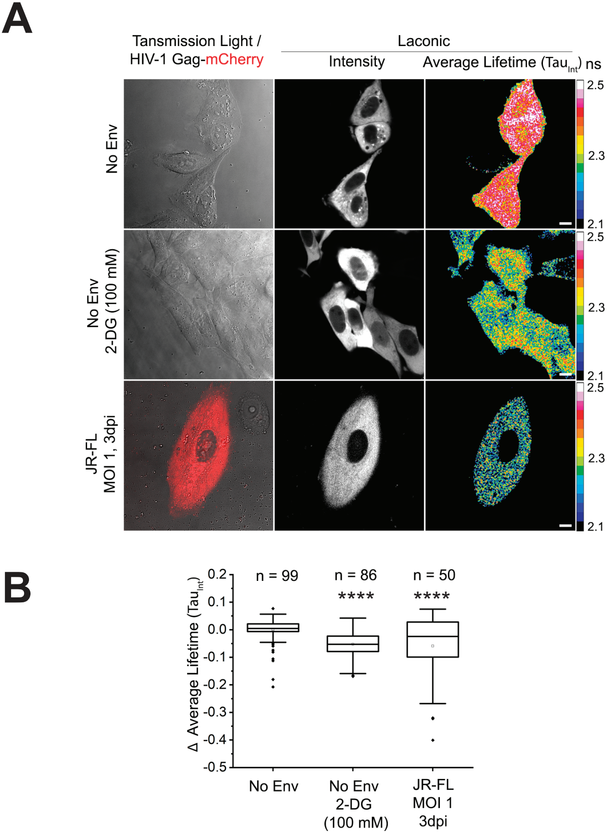

Appendix Figure S3

Appendix Figure S3. Single-cell lactate level in HIV-1 infected and uninfected TZM-bl cells. Panel A) micrograph showing TZM-bl cells expressing Laconic FRET-based biosensor infected with HIV-1 Gag-mCherry pseudoviruses (No Env or JR-FL Env, as indicated), in the absence or presence of the glycolysis inhibitor D-deoxy D-glucose (2-DG). Left column: merge of transmission light image and Gag-mCherry fluorescence showing TZM-bl cells negative (-mCherry) or positive for HIV-1 infection (+mCherry). Middle column: intensity image of Laconic fluorescence. Right column: pixel-by-pixel average lifetime (TauInt) image of Laconic expressing cells represented in 16-colors Look-up Table (LUT). Calibration color bar shows values from 2.1 to 2.5 ns. Scale bar = 10 µm. Panel B) Box and whisker plot showing results from individual TZM-bl cells from 3 independent experiments. Total number of cells analyzed per condition is indicated above each box. Results were analyzed by one-way ANOVA and Sidak post-hoc test **** $p < 0.0001$.

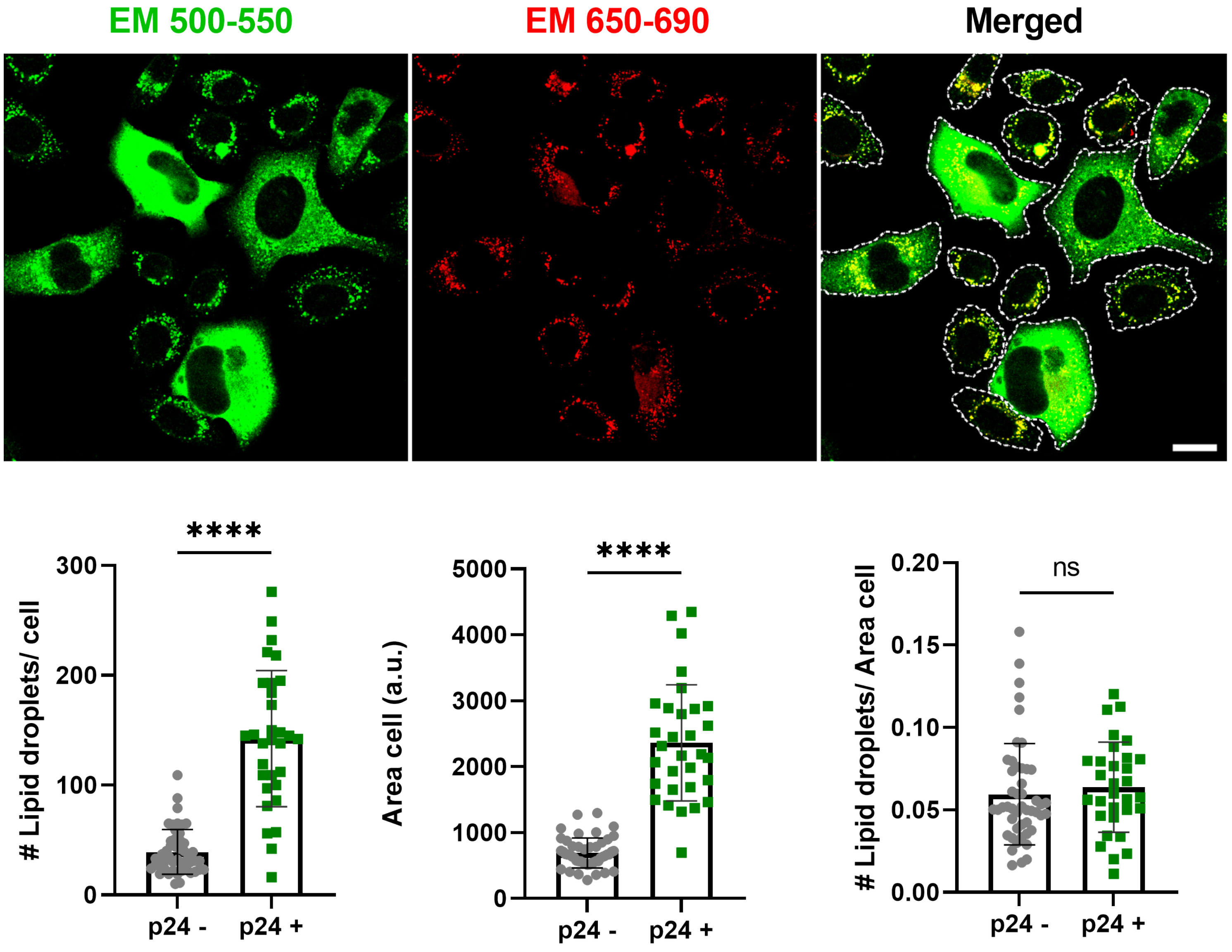

Appendix Figure S4

Appendix Figure S4. Single-cell lipid droplet content in HIV-1 infected and uninfected TZM-bl cells. Panel A) Micrograph showing TZM-bl cells expressing HIV-1 p24 3 days post-infection (Alexa 488 positive cells; emission 500 nm -550 nm, left column) and stained with Nile Red dye for lipid droplet detection (emission 650 nm – 690 nm, middle column). Merge of green and red channels is shown on the right column. Dashed lines indicate the cytoplasm contour of each cell. Scale bar = 20µm. Panels B-D) Scatter plots showing the comparative analysis of infected (A488 positive) vs non-infected cells (A488 negative) cells. Each dot represents the value obtained for a single cell. Bars represent the mean and error bars the standard deviation of at least 30 cells per condition. (B) Quantification of the absolute number of lipid droplets per cell. (C) Quantification of the size (area) of individual cells. (D) Quantification of the number of lipid droplets detected per cell normalized by the size of each cell. Results were analysed using unpaired t-test. **** $p < 0.0001$.

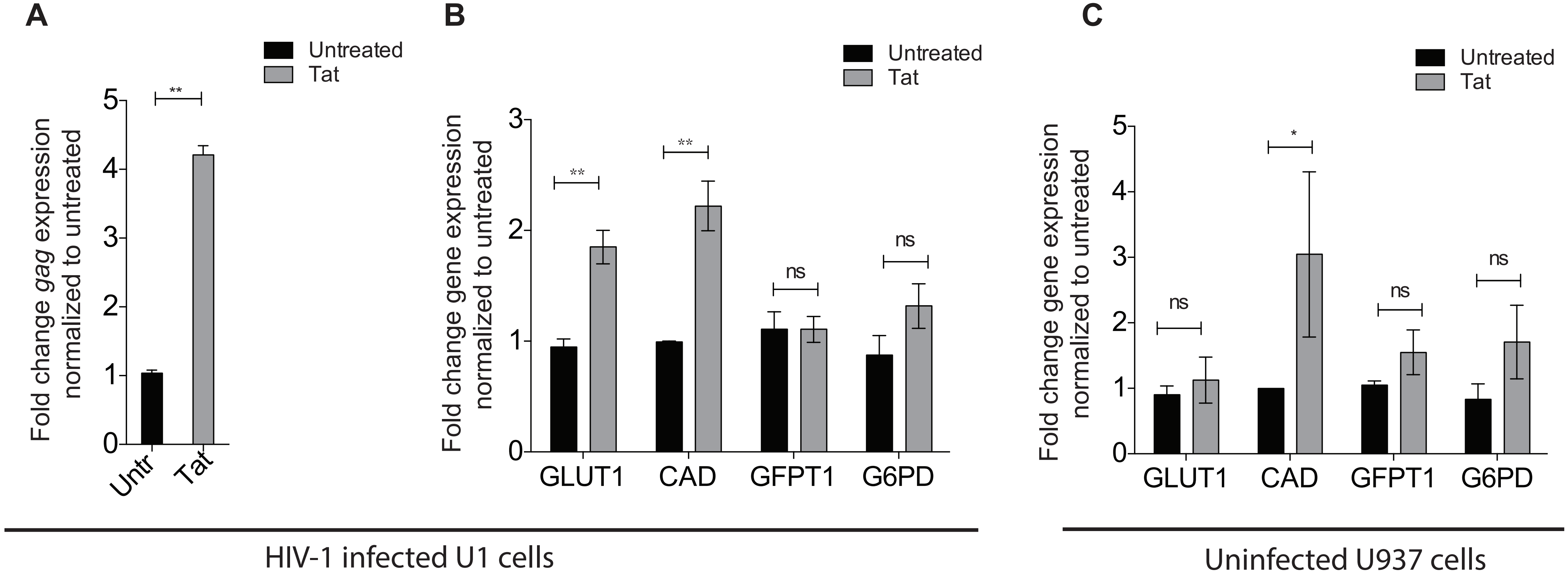

Appendix Figure S5

Appendix Figure S5. Viro-metabolic effects of exogenous Tat administration in HIV-1 infected and uninfected cells. The HIV-1 infected (U1, Panels A,B) and uninfected (U937, Panel C) cell lines, both deficient for Tat signaling, were left untreated or treated with HIV subtype-B Tat protein (800 ng/ml) for 72 h. Panel A. HIV-1 reactivation as measured by qPCR of *gag* expression in infected cells. Panels B,C. Expression of key metabolic genes of glycolysis (*GLUT1*), the pentose phosphate pathway (*G6PD*), pyrimidine biosynthesis/glutamate metabolism (*CAD*) and hexosamine biosynthesis/glutamate metabolism (*GFPT1*) as measured by qPCR in both infected (B) and uninfected cells (C). Fold change variations over the untreated control were calculated using the 2-ΔΔCT method as in (Livak & Schmittgen, 2001). Results are expressed as mean ± SD and are representative of data from two independent experiments (N=2). Results were analyzed by unpaired t-test with Welch's correction (A) or two-way ANOVA followed by Bonferroni's multiple comparison test (B,C). *$p<0.05$; **$p<0.01$.

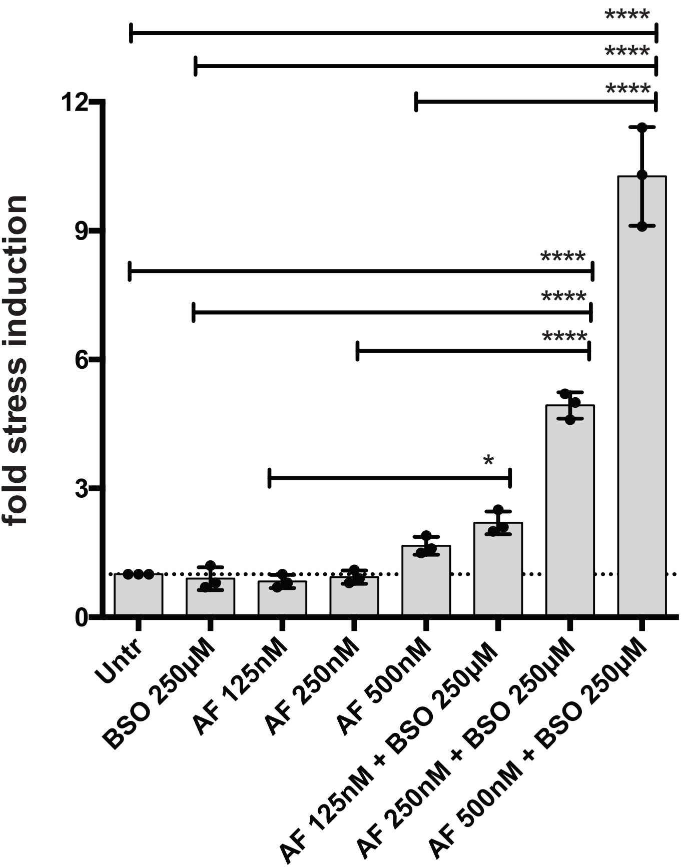

Appendix Figure S6

Appendix Figure S6. Auranofin (AF) and buthionine sulfoximine (BSO) co-treatment induces synergistic oxidative stress in U1 cells. U1 cells expressing the redox sensor Grx1-roGFP2 (Bhaskar *et al*, 2015) were exposed to various combinations of AF and/or BSO and the roGFP2 ratio (405/488 nm) was measured 24 h post-treatment. Fold change was calculated by dividing the roGFP2 ratios under treated conditions with the untreated cells. Data (mean ± SD of three experiments) were analyzed by One Way ANOVA followed by Tukey's post-test (only comparisons between matching drug dosages are shown for simplicity´s sake).
* $p < 0.05$, **** $p < 0.0001$.

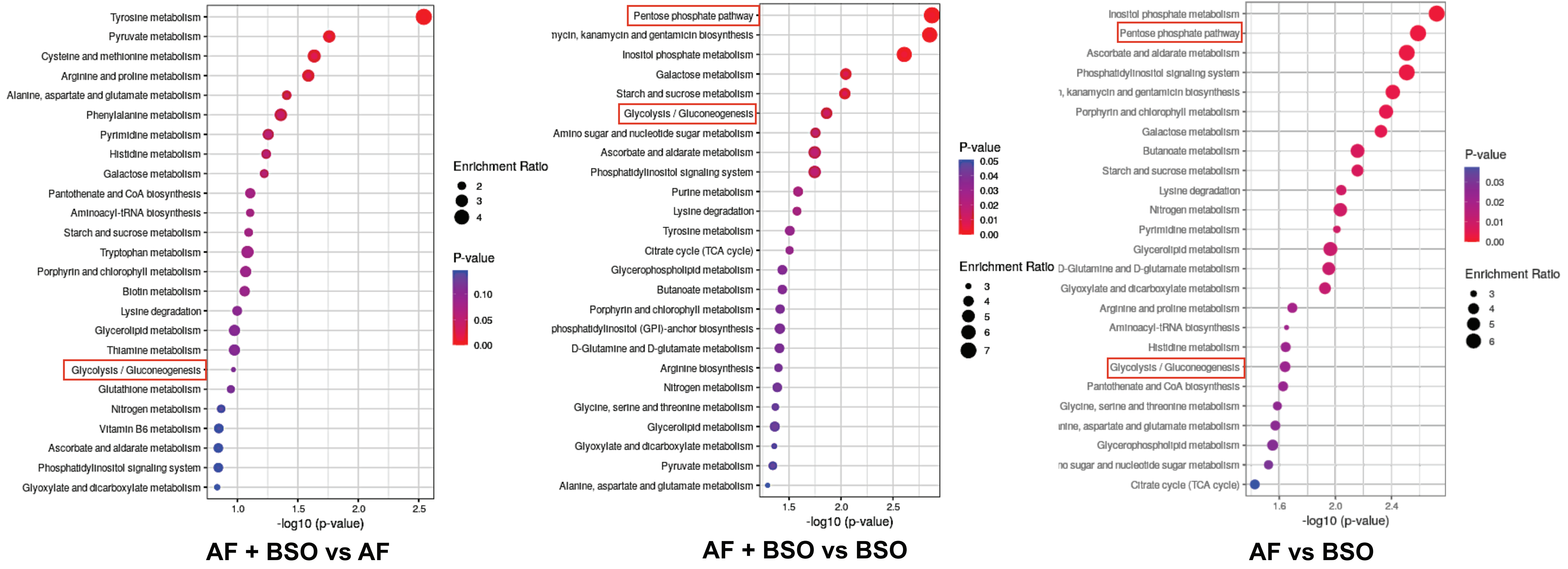

Appendix Figure S7

Appendix Figure S7. Modulation of glycolytic metabolites in cells of PLWH treated with auranofin (AF) and/or buthionine sulfoximine (BSO). Peripheral blood mononuclear cells (PBMC), isolated from total blood of PLWH, were treated with auranofin AF (500 nM), BSO (250 μM) or a combination of the two, for 24 h. PBMC donors were selected from PLWH enrolled in trial NCT02961829 (Diaz *et al*, 2019). Cells were subjected to metabolomic analysis and the top enriched pathways were ordered according to p values obtained with Q statistics for metabolic datasets performed with Globaltest (MetaboAnalyst) (Xia *et al*, 2009). Number of donors = 6.

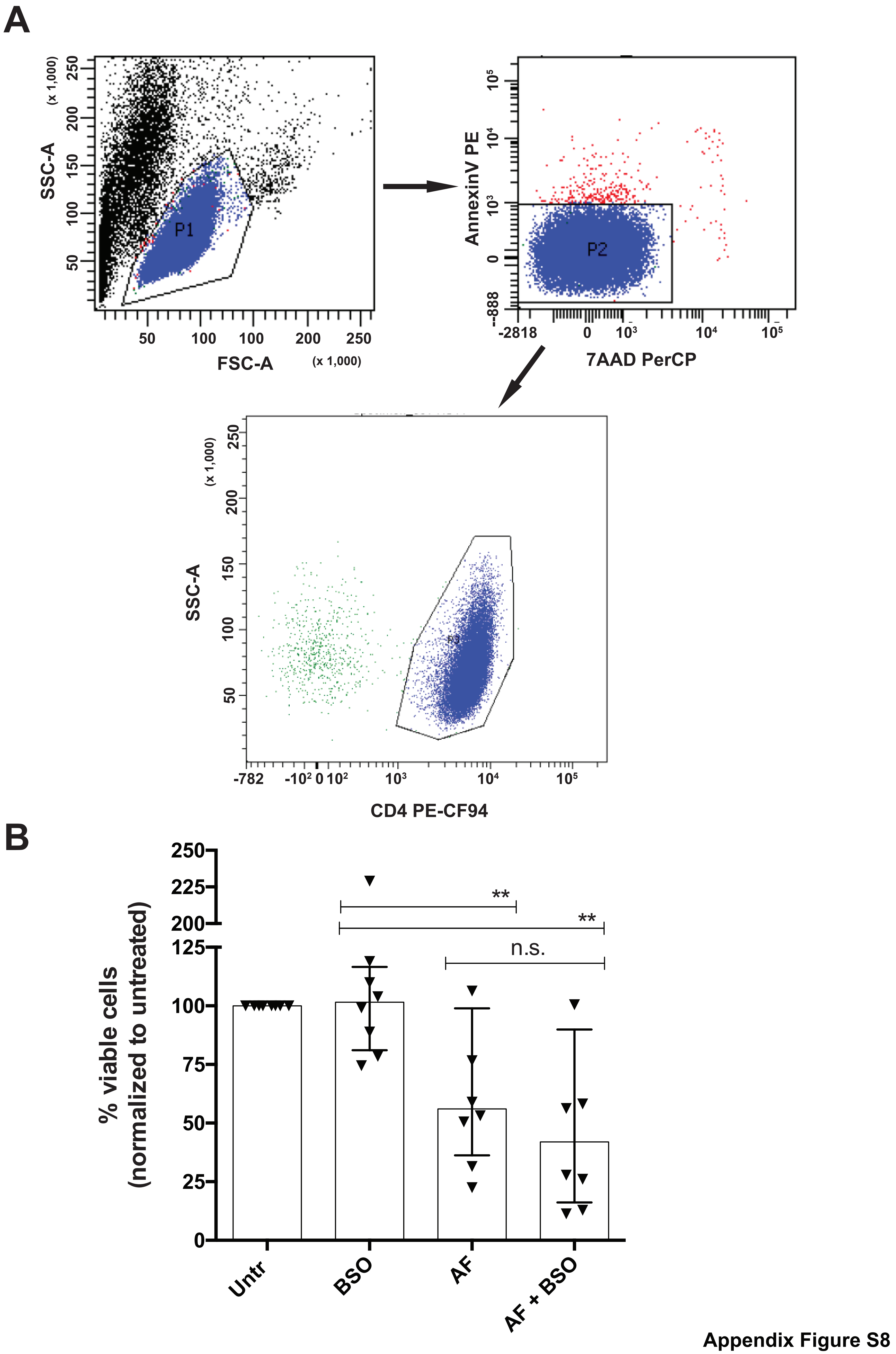

Appendix Figure S8

Appendix Figure S8. Gating strategy and viability of CD4$^+$ T-cells of PLWH under ART left untreated or treated with auranofin (AF) and/or buthionine sulfoximine (BSO). CD4+ T-cells were treated with auranofin AF (500 nM), BSO (250 μM) or a combination of the two, for 24 h. Viable CD4$^+$ T-cells were sorted according to the gating strategy shown in Panel A. Viability data are shown as mean ± SD (Panel B). Data were analyzed by repeated measures one-way ANOVA followed by Tukey's post-test ** $p < 0.01$.

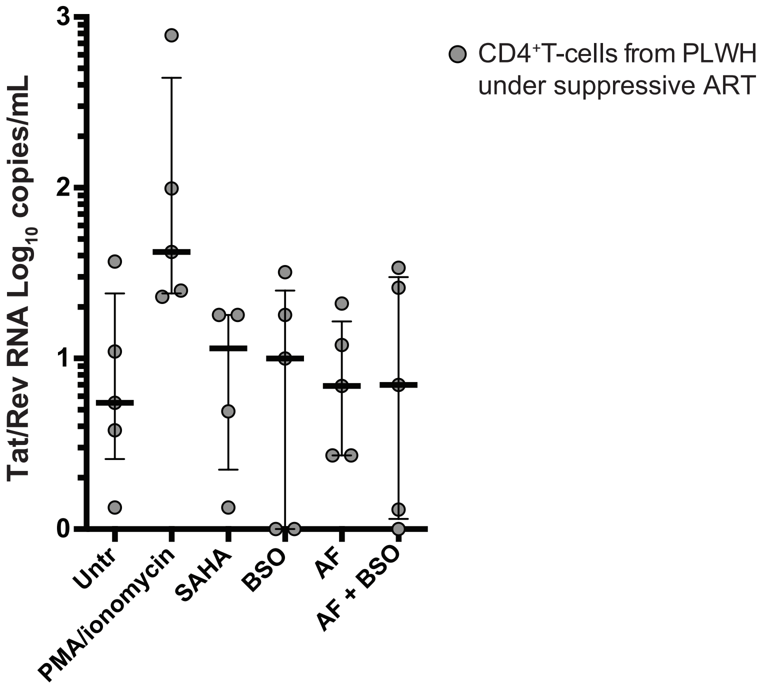

Appendix Figure S9

Appendix Figure S9. HIV-1 reactivation in CD4$^+$ T-cells of PLWH under ART following treatment with auranofin (AF) and buthionine sulfoximine (BSO). CD4$^+$ T-cells were isolated from total blood of PLWH under suppressive ART and left untreated or treated with AF (500 nM), BSO (250 μM) or a combination of the two for 48 h. Viral reactivation was measured by Tat/rev Induced Limiting Dilution Assay [TILDA (CD4$^+$ T-cells of PLWH)] (Procopio *et al*, 2015). Data are expressed as medians and interquartile ranges.
